# Peptidomic Approach for the Identification of Peptides with Potential Antioxidant and Anti-Hyperthensive Effects Derived From Asparagus By-Products

**DOI:** 10.3390/molecules24193627

**Published:** 2019-10-08

**Authors:** Carmela Maria Montone, Riccardo Zenezini Chiozzi, Nicola Marchetti, Andrea Cerrato, Michela Antonelli, Anna Laura Capriotti, Chiara Cavaliere, Susy Piovesana, Aldo Laganà

**Affiliations:** 1 Dipartimento di Chimica, Sapienza Università di Roma, Piazzale Aldo Moro 5, 00185 Rome, Italy; carmelamaria.montone@uniroma1.it (C.M.M.); andrea.cerrato@uniroma1.it (A.C.); michela.antonelli@uniroma1.it (M.A.); chiara.cavaliere@uniroma1.it (C.C.); susy.piovesana@uniroma1.it (S.P.); aldo.lagana@uniroma1.it (A.L.); 2 Biomolecular Mass Spectrometry and Proteomics, Bijvoet Center for Biomolecular Research and Utrecht Institute for Pharmaceutical Sciences, University of Utrecht, Padualaan 8, 3584 CH Utrecht, The Netherlands; riccardo.zenezini@uniroma1.it; 3 Netherlands Proteomics Center, Padualaan 8, 3584 CH Utrecht, The Netherlands; 4 Dipartimento di Chimica e Scienze Farmaceutiche, Università di Ferrara, Via Luigi Borsari 46, 44121 Ferrara, Italy; mrcncl@unife.it; 5 CNR NANOTEC, Campus Ecotekne, Università del Salento, Via Monteroni, 73100 Lecce, Italy

**Keywords:** ACE-inhibitory peptides, antioxidant peptides, asparagus by-products, chromatographic purification, high resolution mass spectrometry, bio-informatic tools

## Abstract

Asparagus waste represents products of great interest since many compounds with high biological value are located in the lower portion of the spears. The extraction of bioactive compounds from asparagus by-products is therefore crucial for the purpose of adding value to these by-products. In this paper, bioactive peptides from asparagus waste were extracted, digested, purified and identified. In particular, Alcalase^®^ was chosen as the enzyme to use to obtain protein hydrolysate due to its low cost and, consequently, the possibility of implementing the method on a large scale. In order to simplify the peptide extract to reach better identification, the hydrolysate was fractionated by reversed-phase chromatography in 10 fractions. Two tests were carried out for antioxidant activity (ABTS-DPPH) and one for antihypertensive activity (ACE). Fractions with a higher bioactivity score were identified by peptidomics technologies and screened for bioactivity with the use of bioinformatics. For ACE-inhibitor activity, two peptides were synthetized, PDWFLLL and ASQSIWLPGWL, which provided an EC_50_ value of 1.76 µmol L^−1^ and 4.02 µmol L^−1^, respectively. For the antioxidant activity, by DPPH assay, MLLFPM exhibited the lowest EC50 value at 4.14 µmol L^−1^, followed by FIARNFLLGW and FAPVPFDF with EC50 values of 6.76 µmol L^−1^ and 10.01 µmol L^−1^, respectively. A validation of the five identified peptides was also carried out. The obtained results showed that peptides obtained from asparagus by-products are of interest for their biological activity and are suitable for being used as functional ingredients.

## 1. Introduction

Asparagus crops have a world production of almost 9 million tons per year. Most of its production comes from China (with 88% of the total production), whereas Italy is the sixth world producer (with 2% of the production), preceded by Peru, Mexico, Germany and Spain (http://faostat3.fao.org/). As a consequence of this intensive production, every year tons of asparagus waste is generated, as only 23.5% of the spear is used for human consumption [1]. Traditionally, the rest of asparagus (about 76%) has been used for animal feeding or low-value by-products. These waste residues cause huge economic and environmental problems, and that is the reason why it is important to discover uses for these waste materials outside the agricultural sector, in order to create sustainable value chains in the farming and processing sectors [2]. In the last decade, increasing attention has been addressed to the recycling of protein or other functional compounds from fruit and vegetable by-products. In the perspective of bio-sustainable development and renewable resource technologies, by-products and waste represent a relatively cheap source of raw material for bioactive molecules extraction [3], reducing the amount of waste and the related costs of disposal and at the same time producing value-added nutritional products [4].

So far, the edible part of asparagus has been the most investigated, showing that the bioactive compounds found in asparagus spears produce beneficial effects on human health, including antitumor and antioxidant activities [5,6], as well as hypocholesterolemic and hepatoprotective effects, hypotensive, renal protective and anti-diabetic properties [7]. However, some recent papers have shown that asparagus by-products are an important source of high-antioxidant compounds, such as soluble peroxidases [8], flavonoids, hydroxycinnamic acids, lignan and steroidal saponin [9], therefore increasing the value of the industrial waste. Thereby, recycling asparagus by-products has gained considerable interest to obtain supplements for nutraceutical, pharmaceutical and cosmetic industries [10]. 

While in the literature many studies reported the presence of bioactive compounds in asparagus, bioactive peptides (BAPs) remain poorly investigated, with just one metabolomics study in which some dipeptides and oligomers with up to five residues were detected in the edible part [7]. Despite proteins, peptides and amino acids being associated with a variety of biological functions in plants, they have received insufficient attention compared to other food sources and other plant metabolites, probably because their detection is difficult due to the low amount present in vegetable samples [11]. The protein content of asparagus is, in fact, around 2 g for every 100 g of product.

To the best of our knowledge, this is the first study specifically related to BAPs performed on asparagus waste products. The purpose of this work was the characterization and identification of BAPs with potential antioxidant and anti-hypertensive activity. A peptidomic approach was carried out in order to achieve this goal. In particular, the isolation and identification of the most active peptide sequences obtained from the digestion of asparagus by-product with Alcalase® has been achieved through the use of preparative reversed phase (RP) liquid chromatography (LC), mass spectrometry (MS) and bioinformatic tools. The in vitro activities and validation of synthesized peptide sequences have also been evaluated.

## 2. Results and Discussion

### 2.1. Fractionation and Purification of ACE-Inhibitory and Antioxidant Peptides From Asparagus By-Products by Preparative RP Chromatography

A bioactivity investigation on the whole asparagus extract provided an antioxidant (AO) average activity of 33% and 32% for 2,2′-azino-bis(3-ethylbenzothiazoline-6-sulphonic acid) (ABTS) and 2,2-Diphenyl-1-picrylhydrazyl (DPPH), respectively, and ACE-inhibitory activity of 70.80%. Data are reported in Supplementary Material Table S1. Those results suggested that the hydrolysate could be a promising source of bioactive peptides. Therefore, the subsequent fractionation and purification was carried out with the purpose of simplifying the hydrolysate mixture and identifying novel bioactive peptides with promising AO and ACE-inhibitory properties.

**Figure 1:**
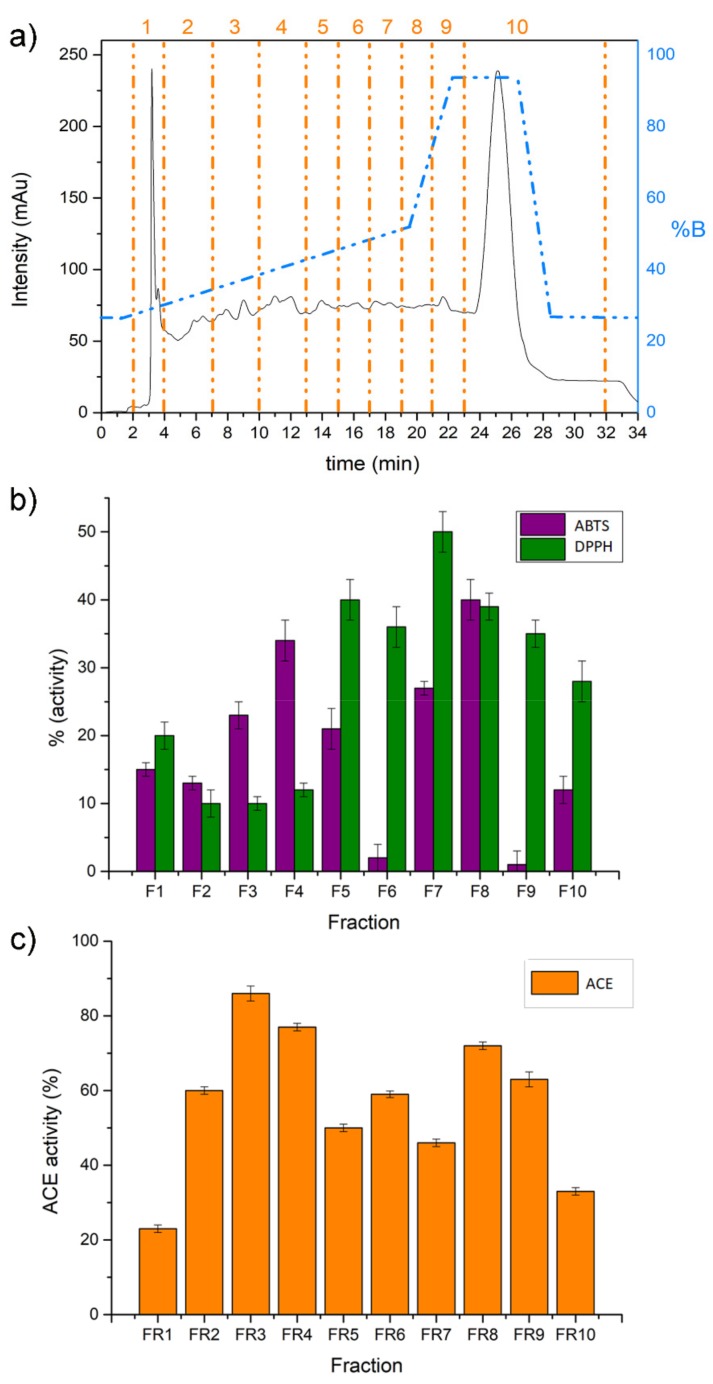
(**a**) Semi-preparative reverse phase high-performance liquid chromatography profile of Alcalase® hydrolysate of asparagus by-product. Fractions (F1–F10) are indicated by vertical dashed lines. The dotted line represents the gradient of solvent B. Bar chart displaying the results of antioxidant (ABTS and DPPH) (**b**) and ACE-inhibitory activity assays on collected fractions (**c**).

Alcalase-generated asparagus hydrolysate was fractionated by a preparative C18 RP chromatography, as shown in Figure 1. Ten fractions were collected (F1–F10) and assayed for the two AO bioactivity assays, namely ABTS and DPPH, and the ACE-inhibitory one. Fractions F4 and F8 were found to possess higher ABTS radical scavenging activity (34 and 40%, respectively), while fractions F5, F7 and F8 had higher DPPH radical scavenging activity (40, 47 and 39%, respectively). F3, F4 and F8 showed the higher ACE inhibitory activity (86, 77 and 72% ACE inhibition, respectively) among all collected fractions (Figure 1 and Supplementary Material Table S1). The peptides with higher biological activity were recovered from the fractions eluted at approximately 30%–50% of MeOH. The majority of the fractions exhibiting ACE inhibition and AO tend to lie near the middle of the chromatogram, an environment with a high abundance of hydrophobic peptides. It is well known from the literature, in fact, that hydrophobic amino acids present higher AO and ACE-inhibitory activities compared to more hydrophilic peptides [12,13]. Hanafi and his co-workers also reported that the majority of ACE inhibitory peptides eluted in the middle or at the end of their RP-HPLC chromatograms [14]. ACE-inhibitor activity was much higher than the AO activity generally for all fractions (data reported in Figure 1 and Supplementary Material S1).

### 2.2. Identification of Peptides by Tandem Mass Spectrometry

The fractions with higher AO and ACE inhibitory activity (F3–F5, F7 and F8) were subjected to nanoHPLC–MS/MS in order to identify the peptides. Peptides identification was performed for the most part by homology against the Swiss-Prot database, which comprises of protein sequences for several green plant species. The homology identification is mandatory for non-model plants when the genome sequence of an organism is not still complete; the *Asparagus officialis* plant falls in this category [15]. 

A total number of 2017 peptides having an area greater than 1E7 were identified and distributed as follows: 1095 peptides were found in F3, 309 in F4, 141 in F5, 398 in F7 and 132 in F8. The complete list of identified peptides for each fraction is reported in Supplementary Material S2. Since most peptides were +2 and +3 charged, Alcalase® has shown to be a very efficient. The peptide charge status implies that peptides can also be very small, which would cause them to be in the initial part of the chromatographic separation (F3 fraction).

Five novel peptide sequences were selected from the five fractions and were synthesized, and their ACE inhibition capacity and AO activity were eventually evaluated in vitro. The selection of specific amino acid sequences for the validation and in vitro assays was carried out by a bioinformatic driven in silico approach. In fact, even if the empirical approach is still the most used, it presents several limitations, especially for a sample with a high complexity. The validation of the amino acid sequence is generally carried out by the synthesis of peptides, which is a very expensive approach. For these reasons, in order to reduce the number of potential candidates, and due to the high number of homology identification, PeptideRanker (http://bioware.ucd.ie) software was employed to assign a score of bioactivity probability to each sequence. For every peptide, PeptideRanker predicts the probability (between 0 and 1) of that peptide being bioactive. The closer the predicted probability is to 1, the more confident PeptideRanker is that the peptide is bioactive; so, in order to restrict the candidates for the synthesis, only peptides possessing a probability score higher than 0.95 were considered. After such filtering of peptide scores, most peptide sequences were rejected and only five peptides were retained in the list of bioactive candidates (Supplementary Information S2). This score is higher than the score usually chosen in analogous papers. The five selected peptides were subjected to in vitro bioassays. The ACE inhibitory and AO activities of peptides and their characteristics are listed in Table 1. 

The peptides contained 7 to 11 amino acid residues with molecular weights ranging from 751 to 1258 Da. The total net charge of the peptides ranged from −1 to 1. Isoelectric points ranged from 3.80 to 9.75, and the hydrophobic ratio of the peptides was found to be in the range of 64% to 100%. Two peptides, namely PDWFLLL and ASQSIWLPGWL, were found to possess ACE inhibitory activity with an EC50 value of 5.74 µmol L^−1^ and 4.02 µmol L^−1^, respectively, while three peptides, namely FAPVPFDF, MLLFPM and FIARNFLLGW, were found to be antioxidants by the DPPH assay. MLLFPM exhibited the lowest EC50 value at 4.14 µmol L^−1^, followed by FIARNFLLGW and FAPVPFDF with EC50 values of 6.76 µmol L^−1^ and 10.01 µmol L^−1^, respectively. The EC50 value obtained in the asparagus sample was compared with the value of captopril, the most widely used antihypertensive drug, which has an EC_50_ of 0.0071 μmol L^−1^ [16]. The EC50 asparagus values were in general still higher, but significantly better compared to the values found in other plant species. For example, ACE-inhibitory peptides obtained from rice, Brewers’ spent grain, soybean and chia (*Salvia hispania*) after Alcalase® digestion possessed EC_50_ values of 18.20 µmol L^−1^, 96.39 µmol L^−1^, 12.3 µmol L^−1^ and 3.97 µmol L^−1^, respectively [17], as well as three peptides, identified and assayed recently in our previous work on cauliflower by-products, that had low ACE-inhibitory values of 17.76 μmol L^−1^, 12.81 μmol L^−1^ and 0.46 μmol L^−1^, respectively [18,19].

Regarding the three AO peptides, they fall exactly in the molecular weight range typical for AO peptides from food (from 500 to 1800 Da), confirming that short peptides possess stronger radical scavenging activity compared to longer sequences [20]. The obtained EC50 values were again comparable to those obtained from already studied plants matrices. Obtained data were in fact quite similar to values found for whole plant extract; however, in this article, specific isolated amino acid sequences have been tested [21]. 

The biological activities of peptides are strictly correlated to the presence of specific amino acids within the peptides sequence. Hydrophobic amino acids and aromatic amino acids at C-terminal play a role in ACE inhibition [14]. The two ACE inhibitory peptides in fact possess a high percentage of hydrophobicity (85.71% and 63.64%). Every synthesized peptide possesses hydrophobic amino acid residues such as Ala, Val, Leu, Ile, Phe, Pro, Trp and Met at C-terminal residue, which is typical when alcalase is used like an enzyme, due to its cleavage preference [22,23]. Alcalase is an alkaline bacterial protease and it has been proven to be one of the best enzymes for the preparation of protein hydrolysates with a high degree of hydrolysis [24]. Alcalase is an endopeptidase, which is able to hydrolyze proteins with a broad specificity for peptide bonds and a preference for hydrophobic side chains [25]. Several studies have shown a relationship with some hydrophobic residues and the antioxidant activity of peptides [26,27]. Ala, Pro and Phe possess scavenging activity for free radicals, whereas Leu and Val, if on the N-terminal, play an important role in the antioxidant activity, with the hypothesis that their aliphatic group promotes interactions with sensitive fatty acids [26,28,29]. Trp and Tyr can work as free radical scavengers [30,31]. Other studies have shown a correlation between antioxidant activity and peptides containing hydrophilic and basic amino acids, such as His-Lys [26,32].

### 2.3. Validation of Bioactive Peptide Sequences

Since false positives are common in peptidomic analysis, the use of synthetic standards of new possible BPs allows certainty of the sequence by comparing retention time, exact mass and fragmentation patterns, and it has the advantage of enabling quantitative analysis of the target peptide in the hydrolysate. Moreover, bioactivity assays on the synthetic standard can help by discerning the effect of the single peptide and, eventually, the synergistic effect in the mixture.

Nevertheless, validation by comparison of a standard and a target peptide in the hydrolysate is seldom performed and partial validation, for which the individual bioactivity of peptides is assayed but the synthesized sequences are not compared to those from the hydrolysate, is much more common [4]. 

The five selected peptides were synthesized and compared with those originally present in the entire hydrolysate. Both the asparagus extract and the standard mixture solution were analyzed by nanoHPLC-MS/MS in order to compare the retention times (Rt) and *m*/*z* values of the standard peptides with that of peptides identified in the asparagus sample. Moreover, bioactivity tests were repeated and performed on the five synthesized standards to evaluate the values of EC50 (Table 2).

Since the *Δm*/*z* was in the range −1.4–1.9 ppm and the ΔRt was within 1.06 min, the match was considered positive. Such retention time driftings are indeed acceptable for nanoHPLC-MS analysis. Retention time drifting is a well-known phenomenon that negatively affects proteomics and metabolomics dataset alignment [33] and can be caused by several factors, such as the conditions and the aging of the chromatographic column and the temperature fluctuations [34,35]. 

## 3. Materials and Methods

### 3.1. Chemicals and Reagents

All chemicals, reagents and organic solvents of the highest grade available were purchased from Sigma-Aldrich (St. Louis, MO, USA) unless otherwise stated. DPPH, ACE from rabbit lung, *N*-Hippuryl-l-histidyl-l-leucine (HHL) and Hippuric acid were purchased from Sigma-Aldrich (St. Louis, MO, USA). 

Deionized water was prepared by an Arium 611 VF system from Sartorius (Göttingen, Germany). Solid Phase Extraction (SPE) C18 cartridges were Bond Elut (Varian, Palo Alto, CA, USA). 

### 3.2. Pant Material, Protein Extraction and Precipitation

Asparagus by-products (*Asparagus officinalis*) were supplied by a local farm located in Emilia Romagna region (Italy). Asparagus by-products were washed with deionized water, chopped with a sharp stainless steel knife in small pieces, lyophilized to reduce the water content (about 80%) and then ground to a fine powder with liquid nitrogen. One g of ground material was extracted with 50 mL of a buffer consisting of tris-50 mmol L^−1^ Tris-HCl (pH 8.8), 15 mmol L^−1^ NaCl and 1% (*w*/*v*) SDS. The samples were placed in an ultrasonic bath for 6 h with intermittent vortexing (1 h) every 30 min. Then they were incubated for 30 min at 100 °C, and finally, the insoluble material was removed by centrifugation at 4 °C for 15 min at 11,000× *g*. The supernatant was transferred into new centrifuge tubes and subjected to a protein precipitation procedure by adding 4 volumes of a solution made up of 10% (*w*/*v*) trichloroacetic acid (TCA) in acetone, as previously described [36]. After the overnight precipitation at −20 °C, the obtained pellet was collected by centrifugation (18,400× *g* for 15 min at 4 °C), washed three times with ice-cold acetone, air-dried and dissolved in 2 mL of 8 mol L^−1^ urea (pH 8.5) in tris-50 mmol L-1 Tris-HCl (pH 8.8). Protein samples were quantified by the Bradford assay using bovine serum albumin (BSA) as the standard and stored at −80 °C until digestion. 

### 3.3. Protein Digestion

After quantification, the asparagus by-product protein pellet was hydrolyzed by Alcalase®, as previously described [18]. Briefly, 150 mg protein aliquot was diluted with 50 mmol L^−1^ Tris-HCl (pH 8.8) to obtain a final urea concentration of 0.8 mol L^−1^. Alcalase was added (1:10, enzyme: protein ratio) and samples were incubated at 60 °C for 4 h. Enzymatic hydrolysis was quenched by decreasing the pH to 2 with trifluoroacetic acid (TFA). The resulting peptide mixture was stored at −20 °C until analysis.

### 3.4. Peptide Solid Phase Extraction 

Hydrolysate sample was purified by solid phase extraction (SPE) on C18 cartridges, previously conditioned with acetonitrile (ACN). After loading, peptides were rinsed with 0.1% TFA aqueous solution, eluted with ACN/ddH_2_O (70/30, *v*/*v*) with 0.1% TFA and then dried in the Speed-Vac SC250 Express (Thermo Savant, Holbrook, NY, USA, 20 Torr to 100 mTorr, 1400 rpm). Samples were reconstituted with 250 μL of 0.1% formic acid (HCOOH) aqueous solution for subsequent chromatographic purification.

### 3.5. Purification of Bioactive Peptides 

Hydrolyzed peptides, using Alcalase®, were purified using an Xbridge BEH preparative C18 5 μm OBD 19 × 250 mm (Waters) connected to a Shimadzu Prominence LC-20A system, including a CBM-20A controller, two LC-20 AP preparative pumps and a DGU-20A3R online degasser. An SPD-20A UV with a preparative cell (0.5 mm) was used as a detector and was set at 214nm. An FRC-10A Shimadzu was employed as an auto collector. Data acquisition was performed by the LabSolution version 5.53 software (Shimadzu, Kyoto, Japan).

**Figure 2:**
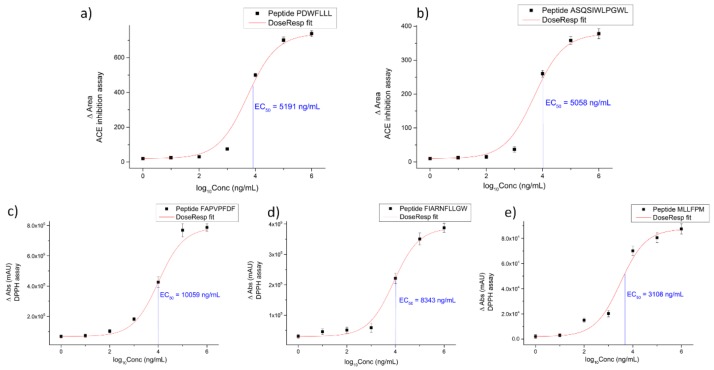
Graphs reporting the EC50 values of the peptides that resulted as bioactive in the in vitro ACE-inhibitory assay (**a**,**b**) and DPPH assay (**c**,**d**,**e**).

The sample was eluted with a flow rate of 17 mL min^−1^ using ddH2O/TFA (99.9/0.1, *v/v*) as phase A and MeOH/TFA (99.9/0.1, *v/v*) as phase B. The gradient started with 1 min of 25% phase B, and then increased to 50% in 19 min; finally, B was increased to 95% in 1 min and maintained constant for 5 min. The column was re-equilibrated for 6 min at 25% B. Ten fractions were collected every 3 min (except for fraction 1 and 12, as shown in Figure 2). Collected peptide fractions (F1–F10) were subjected to bioactivity tests to identify the most active ones.

### 3.6. Bioactivity Assays

Samples have been tested for ACE-inhibitory and AO activities. In particular, 1 mg of the whole hydrolysate was tested with the two aforementioned assays. Moreover, the assays were again carried out on the fractions obtained by RP preparative purification. 

For the study of ACE-inhibitory and AO-DPPH activity, the same protocol as in our previous work was used [37]. Briefly, ACE-inhibitory assay was performed in 50 mmol L^−1^ Tris buffer (pH 8.3) containing 300 mmol L^−1^ NaCl. The starting assay volume was 50 μL of the substrate (5 mmol L^−1^), 50 μL of ACE solution with 1 mU of declared enzyme activity and 50 μL of assay sample. Then an incubation step at 37 °C for 90 min occurred. The reaction was quenched by adding 250 μL of 1 mol L^−1^ HCl, and the final hippuric acid was extracted with 1.5 mL of ethyl acetate, and centrifuged for 15 min at 2500× *g* and 25 °C. After drying down the organic layer, the hippuric acid was dissolved in 3 mL ddH2O. Therefore, the absorbance was measured at 228 nm. For the AO-DPPH assay, the DPPH solution was diluted with methanol. Asparagus peptide fractions were reconstituted with 2 mL of water mixed with 2 mL of methanol solution containing 0.125 mmol L^−1^ DPPH radicals. The sample was kept for 60 min in the dark, and then the absorbance was determined at 517 nm (t = 0 min) and after 60 min (t = 60) by a UV-visible spectrophotometer (V-530; Jasco, Easton, Oklahoma City, OK, USA).

For the ABTS AO assay, the protocol from Re and coworkers [37] was carried out with some modifications. ABTS was dissolved in water to a 7 mmol L^−1^ concentration. The ABTS radical cation was produced by reacting ABTS stock solution with 2.45 mmol L^−1^ potassium persulfate (final concentration) and allowing the mixture to stand in the dark at RT for 12–16 h. The solution was diluted 1:20 (*v*/*v*) before use. One milligram of peptides was solubilized in 2 mL of water and added to 1 mL of ABTS solution. The absorbance was measured at 745 nm.

### 3.7. NanoHPLC-MS/MS Analysis

The most active fractions obtained the RP preparative chromatographic purification were analyzed by nanoHPLC coupled to MS/MS. The analysis was performed on a Dionex Ultimate 3000 (Dionex Corporation, Sunnyvale, CA, USA). Peptide mixtures were enriched on a 300 μm ID × 5 mm Acclaim PepMap 100 C18 (5 μm particle size, 100 Å pore size) precolumn (Dionex Corporation Sunnyvale, CA, USA), employing a premixed mobile phase made up of ddH_2_O/ACN 98/2 (*v*/*v*), containing 0.1% (*v*/*v*) TFA, at a flow-rate of 10 μL min^−1^. Then, peptide mixtures were separated by RP chromatography using an LC system equipped with a 25 cm long fused silica nanocolumn, 75 μm ID, in-house packed with Acclaim-C18 2.2 μm microparticles and outlet Kasil frit. The LC gradient was optimized to detect the largest set of peptides using ddH_2_O/HCOOH (99.9/0.1, *v*/*v*) as mobile phase A and ACN/HCOOH (99.9/:0.1, *v*/*v*) as mobile phase B. After an isocratic step at 2% B for 5 min, B was linearly increased to 5% within 2 min and then to 35% within 90 min; afterward, phase B was increased to 80% within the following 3 min. Then, phase B was maintained at 80% for 20 min to rinse the column. Finally, B was lowered to 2% in 1 min and the column was re-equilibrated for 40 min.

The nanoLC system was coupled to an LTQ-Orbitrap XL hybrid mass spectrometer (Thermo Fisher Scientific, Bremen, Germany) via a nanoelectrospray ion source, operated in positive ionization mode, with spray and capillary voltage set at 2.90 kV and 42 V, respectively, and capillary temperature set at 180 °C. Full MS spectra were acquired in profile mode in the *m*/*z* range 350–1800 in the Orbitrap with resolution set at 60,000, whereas the data-dependent MS/MS scan of the five most intense monoisotopic peaks in the spectra (top five strategy) was operated with collision-induced dissociation activation at low resolution in the LTQ. Rejection of singly charged and unassigned charge states was enabled. All MS/MS spectra were collected using a normalized collision energy of 35%, and an isolation window of 2 *m*/*z*. Ion trap and Orbitrap maximum ion injection times were set to 1000 and 200 ms, respectively. Automatic gain control was used to prevent overfilling of the ion traps and was set to 5 × 10^5^.

### 3.8. Database Search and Peptide Identification

All raw files from Xcalibur software (version 2.2 SP1.48, Thermo Fisher Scientific) were submitted to Proteome Discoverer software (version 1.3, Thermo Scientific) with the Mascot search engine (v.2.3, Matrix Science) for peptide/protein identification. The searches were performed against the proteome of *Asparagus officinalis L* species from Uniprot (http://www.uniprot.org/), as previously described [34], but no enzyme specificity was set. The potential bioactive peptides were subsequently filtered using three parameters: a Mascot-score larger than 30, an area of the integrated peak larger than 10^7^ and the score of PeptideRanker larger than 0.95. The potential peptide candidates were searched against the BIOPEP database (http://www.uwm.edu.pl/biochemia/index.php/en/biopep) and PepBank (http://pepbank.mgh.harvard.edu/) to determine their novelty.

### 3.9. Peptide Synthesis

The selected peptide sequences, namely having a score higher than 0.95 (PDWFLLL, FAPVPFDF, MLLFPM, FIARNFLLGW and ASQSIWLPGWL) were synthesized by Life Technologies Europe (Thermo Scientific, Bremen, Germany) at the highest available purity (90%). Peptide standards were reconstituted in the bioassay specific buffer and tested to calculate the EC50 values; all fitting calculations were done using Origin 9.0. Peptide standards were reconstituted with 0.1% HCOOH aqueous solution. Twenty microliters of the mix solution were injected in the nanoHPLC coupled to the Orbitrap mass spectrometer.

## 4. Conclusions

In this study, for the first time, bioactive peptides from asparagus waste were identified. The optimized conditions of extraction and digestion with a low-cost commercial enzyme allow for large-scale implementation. Furthermore, many studies of literature analyze only the bioactivity of the various fractions coming from the chromatographic separation. In this case, all the specific amino acid sequences of the chromatographic fractions with high bioactivity were studied. A bioinformatic software, based on a neural algorithm able to assign a probability score, was used to reduce the number of bioactive candidates. Among these, five peptides, possessing a probability score higher than 0.95, were synthesized and in vitro assayed and validated. Three novel AO and two novel ACE-inhibitory peptides were therefore extracted, purified and identified. Given the good values of biological activity, they appear suitable candidates for future application in the nutraceutical field.

The obtained results, nevertheless, demonstrate the suitability of the preparation process for bioactive hydrolysates from asparagus wastes. In further studies, the impact of digestion as well as the intestinal adsorption needs to be evaluated for the use of identified peptides in the nutraceutical field.

## Figures and Tables

**Table 1:** Synthesized peptide sequences derived from asparagus by-products Alcalase hydrolysate and their properties.

Peptide Sequence	Fraction	Peptide Ranker Score	Molecular Weight (g mol^−1^)	Net Charge at pH 7.4	Calculated Isoelectric Point *	GRAVY **	Biological Activity	Bioactivity Assay	EC50 value ± SD (µmol L^−1^)
PDWFLLL	8	0.97	903.09	−1	3.8	1.17	ACE	ACE Inhibition Assay	5.74 ± 2.35
FAPVPFDF	7	0.96	939.07	−1	3.8	0.96	AO	DPPH	10.71 ± 4.86
MLLFPM	7	0.95	751.01	0	5.28	2.1	AO	DPPH	4.14 ± 1.81
FIARNFLLGW	7	0.95	1236.48	1	9.75	1.02	AO	DPPH	6.76 ± 4.54
ASQSIWLPGWL	3	0.95	1257.45	0	5.57	0.45	ACE	ACE Inhibition Assay	4.02 ± 1.79

* Isoelectric points are obtained from the Compute pI/mW tool at https://web.expasy.org/compute_pi/. ** GRAVY values are obtained from http://www.gravy-calculator.de/index.

**Table 2:** Sequences of the five synthetized bioactive peptides with the corresponding observed [MH]^+^, *m*/*z* values and retention times (Rt) for the standard peptides and for the asparagus by-product sample. The difference between the standard peptide and the asparagus by-product peptides is also reported.

Amino Acid Sequence	[MH] + (Da)	Standard		Asparagus Extracts		Difference	
		*m*/*z*	R_t_ (min)	*m*/*z*	R_t_ (min)	*Δm*/*z* (ppm)	ΔR_t_ (min)
PDWFLLL	903.4921	452.2456	62.02	452.2461	60.96	1.5	1.06
FAPVPFDF	939.4553	470.2268	55.31	470.2277	55.83	1.3	−0.52
MLLFPM	751.3954	376.1991	44.71	376.1977	43.86	−1.4	0.85
FIARNFLLGW	1236.7000	618.8509	45.92	618.8500	45.05	1.9	0.87
ASQSIWLPGWL	1257.6615	629.3303	36.24	629.3308	36.61	1.2	−0.37

## Supplementary Materials

The following are available online at https://www.mdpi.com/1420-3049/24/19/3627/s1, Table S1: ACE-inhibitor and AO activity percentage of 10 collected fractions, Table S2: peptide identifications and PeptideRanker score.

## Abbreviation

ACE	angiotensin-converting enzyme
AO	antioxidant
BAPs	bioactive peptides
BSA	bovin serum albumin
DPPH	2,2-Diphenyl-1-picrylhydrazyl
DTT	dithiothreitol
EDTA	ethylenediaminetetraacetic acid
HHL	*N*-Hippuryl-l-histidyl-l-leucine
IAA	iodacetamide
SDS	sodium dodecyl sulfate
SPE	solid phase extraction
RP	reversed phase
TCA	trichloroacetic acid
TFA	trifluoroacetic acid

## References

[1] Zhang H., Birch J., Yang H., Xie C., Kong L., Dias G., Bekhit A.E.-D. (2018). Effect of solvents on polyphenol recovery and antioxidant activity of isolates of Asparagus Officinalis roots from Chinese and New Zealand cultivars. Int. J. Food Sci. Technol..

[2] Herrero M., del Pilar Sánchez-Camargo A., Cifuentes A., Ibáñez E. (2015). Plants, seaweeds, microalgae and food by-products as natural sources of functional ingredients obtained using pressurized liquid extraction and supercritical fluid extraction. TrAC Trends Anal. Chem..

[3] Montone C.M., Capriotti A.L., Cavaliere C., la Barbera G., Piovesana S., Zenezini Chiozzi R., Laganà A. (2018). Peptidomic strategy for purification and identification of potential ACE-inhibitory and antioxidant peptides in Tetradesmus obliquus microalgae. Anal. Bioanal. Chem..

[4] Piovesana S., Capriotti A.L., Cavaliere C., La Barbera G., Montone C.M., Zenezini Chiozzi R., Laganà A. (2018). Recent trends and analytical challenges in plant bioactive peptide separation, identification and validation. Anal. Bioanal. Chem..

[5] Fuentes Alventosa J.M., Moreno Rojas J.M. (2015). Bioactive Compounds in Asparagus and Impact of Storage and Processing. Processing and Impact on Active Components in Food.

[6] Lee J.W., Lee J.H., Yu I.H., Gorinstein S., Bae J.H., Ku Y.G. (2014). Bioactive Compounds, Antioxidant and Binding Activities and Spear Yield of Asparagus officinalis L.. Plant Foods Hum. Nutr..

[7] Jiménez-Sánchez C., Lozano-Sánchez J., Rodríguez-Pérez C., Segura-Carretero A., Fernández-Gutiérrez A. (2016). Comprehensive, untargeted, and qualitative RP-HPLC-ESI-QTOF/MS2 metabolite profiling of green asparagus (Asparagus officinalis). J. Food Compos. Anal..

[8] Jaramillo-Carmona S., Lopez S., Vazquez-Castilla S., Rodriguez-Arcos R., Jimenez-Araujo A., Guillen-Bejarano R. (2013). Asparagus Byproducts as a New Source of Peroxidases. J. Agric. Food Chem..

[9] Fuentes-Alventosa J.M., Jaramillo-Carmona S., Rodríguez-Gutiérrez G., Guillén-Bejarano R., Jiménez-Araujo A., Fernández-Bolaños J., Rodríguez-Arcos R. (2013). Preparation of bioactive extracts from asparagus by-product. Food Bioprod. Process..

[10] Fan R., Yuan F., Wang N., Gao Y., Huang Y. (2015). Extraction and analysis of antioxidant compounds from the residues of Asparagus officinalis L.. J. Food Sci. Technol..

[11] Farrokhi N., Whitelegge J.P., Brusslan J.A. (2008). Plant peptides and peptidomics. Plant Biotechnol. J..

[12] Daskaya-Dikmen C., Yucetepe A., Karbancioglu-Guler F., Daskaya H., Ozcelik B. (2017). Angiotensin-I-Converting Enzyme (ACE)-Inhibitory Peptides from Plants. Nutrients.

[13] Zou T.-B., He T.-P., Li H.-B., Tang H.-W., Xia E.-Q. (2016). The Structure-Activity Relationship of the Antioxidant Peptides from Natural Proteins. Molecules.

[14] Hanafi M.A., Hashim S.N., Chay S.Y., Ebrahimpour A., Zarei M., Muhammad K., Abdul-Hamid A., Saari N. (2018). High angiotensin-I converting enzyme (ACE) inhibitory activity of Alcalase-digested green soybean (Glycine max) hydrolysates. Food Res. Int..

[15] Capriotti A.L., Cavaliere C., Foglia P., Piovesana S., Samperi R., Stampachiacchiere S., Laganà A. (2013). Proteomic platform for the identification of proteins in olive (Olea europaea) pulp. Anal. Chim. Acta.

[16] Tsai J.-S., Chen T.-J., Pan B.S., Gong S.-D., Chung M.-Y. (2008). Antihypertensive effect of bioactive peptides produced by protease-facilitated lactic acid fermentation of milk. Food Chem..

[17] Lee S.Y., Hur S.J. (2017). Antihypertensive peptides from animal products, marine organisms, and plants. Food Chem..

[18] Montone C.M., Capriotti A.L., Cavaliere C., La Barbera G., Piovesana S., Zenezini Chiozzi R., Laganà A. (2018). Characterization of antioxidant and angiotensin-converting enzyme inhibitory peptides derived from cauliflower by-products by multidimensional liquid chromatography and bioinformatics. J. Funct. Foods.

[19] Caliceti C., Capriotti A.L., Calabria D., Bonvicini F., Zenezini Chiozzi R., Montone C.M., Piovesana S., Zangheri M., Mirasoli M., Simoni P. (2019). Peptides from Cauliflower By-Products, Obtained by an Efficient, Ecosustainable, and Semi-Industrial Method, Exert Protective Effects on Endothelial Function. Oxid. Med. Cell. Longev..

[20] Zhang M., Mu T.-H. (2017). Identification and characterization of antioxidant peptides from sweet potato protein hydrolysates by Alcalase under high hydrostatic pressure. Innov. Food Sci. Emerg. Technol..

[21] Mensor L.L., Menezes F.S., Leitão G.G., Reis A.S., Santos T.C.D., Coube C.S., Leitão S.G. (2001). Screening of Brazilian plant extracts for antioxidant activity by the use of DPPH free radical method. Phyther. Res..

[22] Lourenço da Costa E., Antonio da Rocha Gontijo J., Netto F.M. (2007). Effect of heat and enzymatic treatment on the antihypertensive activity of whey protein hydrolysates. Int. Dairy J..

[23] Sae-Leaw T., Karnjanapratum S., O’Callaghan Y.C., O’Keeffe M.B., FitzGerald R.J., O’Brien N.M., Benjakul S. (2017). Purification and identification of antioxidant peptides from gelatin hydrolysate of seabass skin. J. Food Biochem..

[24] Benjakul S., Morrisseyl M.T. (1997). Protein Hydrolysates from Pacific Whiting Solid Wastes. J. Agric. Food Chem..

[25] Graycar T.P., Bott R.R., Power S.D., Estell D.A. (2013). Subtilisins. Handb. Proteolytic Enzymes.

[26] Chen H.-M., Muramoto K., Yamauchi F., Fujimoto K., Nokihara K. (1998). Antioxidative Properties of Histidine-Containing Peptides Designed from Peptide Fragments Found in the Digests of a Soybean Protein. J. Agric. Food Chem..

[27] Li B., Chen F., Wang X., Ji B., Wu Y. (2007). Isolation and identification of antioxidative peptides from porcine collagen hydrolysate by consecutive chromatography and electrospray ionization–mass spectrometry. Food Chem..

[28] Elias R.J., Kellerby S.S., Decker E.A. (2008). Antioxidant Activity of Proteins and Peptides. Crit. Rev. Food Sci. Nutr..

[29] Park P.-J., Jung W.-K., Nam K.-S., Shahidi F., Kim S.-K. (2001). Purification and characterization of antioxidative peptides from protein hydrolysate of lecithin-free egg yolk. J. Am. Oil Chem. Soc..

[30] Pihlanto A. (2006). Antioxidative peptides derived from milk proteins. Int. Dairy J..

[31] Rajapakse N., Mendis E., Byun H.-G., Kim S.-K. (2005). Purification and in vitro antioxidative effects of giant squid muscle peptides on free radical-mediated oxidative systems. J. Nutr. Biochem..

[32] Hattori M., Yamaji-Tsukamoto K., Kumagai H., Feng Y., Takahashi K. (1998). Antioxidative Activity of Soluble Elastin Peptides. J. Agric. Food Chem..

[33] Liu Q., Cobb J.S., Johnson J.L., Wang Q., Agar J.N. (2014). Performance comparisons of nano-LC systems, electrospray sources and LC-MS-MS platforms. J. Chromatogr. Sci..

[34] Wandy J., Daly R., Breitling R., Rogers S. (2015). Incorporating peak grouping information for alignment of multiple liquid chromatography-mass spectrometry datasets. Bioinformatics.

[35] Piovesana S., Capriotti A.L., Cavaliere C., La Barbera G., Samperi R., Zenezini Chiozzi R., Laganà A. (2015). Peptidome characterization and bioactivity analysis of donkey milk. J. Proteomics.

[36] Zenezini Chiozzi R., Capriotti A.L., Cavaliere C., La Barbera G., Piovesana S., Laganà A. (2016). Identification of three novel angiotensin-converting enzyme inhibitory peptides derived from cauliflower by-products by multidimensional liquid chromatography and bioinformatics. J. Funct. Foods.

[37] Re R., Pellegrini N., Proteggente A., Pannala A., Yang M., Rice-Evans C. (1999). Antioxidant activity applying an improved ABTS radical cation decolorization assay. Free Radic. Biol. Med..

